# Can the supplementation of a digestive enzyme complex offer a solution for common digestive problems?

**DOI:** 10.1186/2049-3258-72-S1-P7

**Published:** 2014-06-06

**Authors:** Thomas Quinten, Jean-Michel Philippart, Thomas De Beer, Stefaan Vervarcke, Mieke Van Den Driessche

**Affiliations:** 1 Metagenics Europe, Edward Vlietinckstraat 20, BE-8400 Oostende, Belgium; 2 Laboratory of Pharmaceutical Process Analytical Technology, Ghent University, B-9000 Ghent, Harelbekestraat 72, Belgium

## Introduction

Proper functioning of the digestive system is imperative to assimilate nutrients, to sustain essential functions in the human body, to increase the bioavailability of nutrients, to minimize the risk of food intolerances, and to reduce the formation of toxins/irritants in the gastrointestinal-tract. Incomplete digestion often results in digestive problems such as bloating, diarrhea, stomach pain and cramps. Physicians often encounter these problems, treatment includes the use of gastroprokinetic drugs and lifestyle changes. The aim of this study was to compare the use of a gastroprokinetic agent with a full spectrum digestive enzyme complex from non-animal origin in relieving common digestive complaints.

## Material and methods

An observational study was performed with 62 volunteers suffering from common digestive problems. All volunteers were eligible for treatment with a gastroprokinetic following anamnesis by a physician. Prior to the start of the study, each volunteer had to complete a validated questionnaire consisting of eight questions addressing the severity of various symptoms related to digestive disorders (0: absent, 1: low, 2: mild, 3: average, 4: severe). Then, patients were randomly assigned to a group receiving domperidone (n=19) [(Motilium®, Janssen-Cilag); dose regimen defined by physician] or an enzyme complex (non-animal origin) (n=43) [(Similase Total®, Metagenics Europe, active between pH 2-12); dose regimen: 1 capsule/meal] and treated for five consecutive days. At the end of the study, volunteers had to complete the same questionnaire and scores were collected. The mean and standard deviation were calculated and a paired two-sample t-tests was performed to investigate if significant differences were seen before and after treatment for each question in each group. In order to calculate differences between the domperidone and Similase group after treatment, unpaired two-sample t-tests were used (α=0.05). The Shapiro-Wilk test was used to assess normality.

## Results

**Figure 1:**
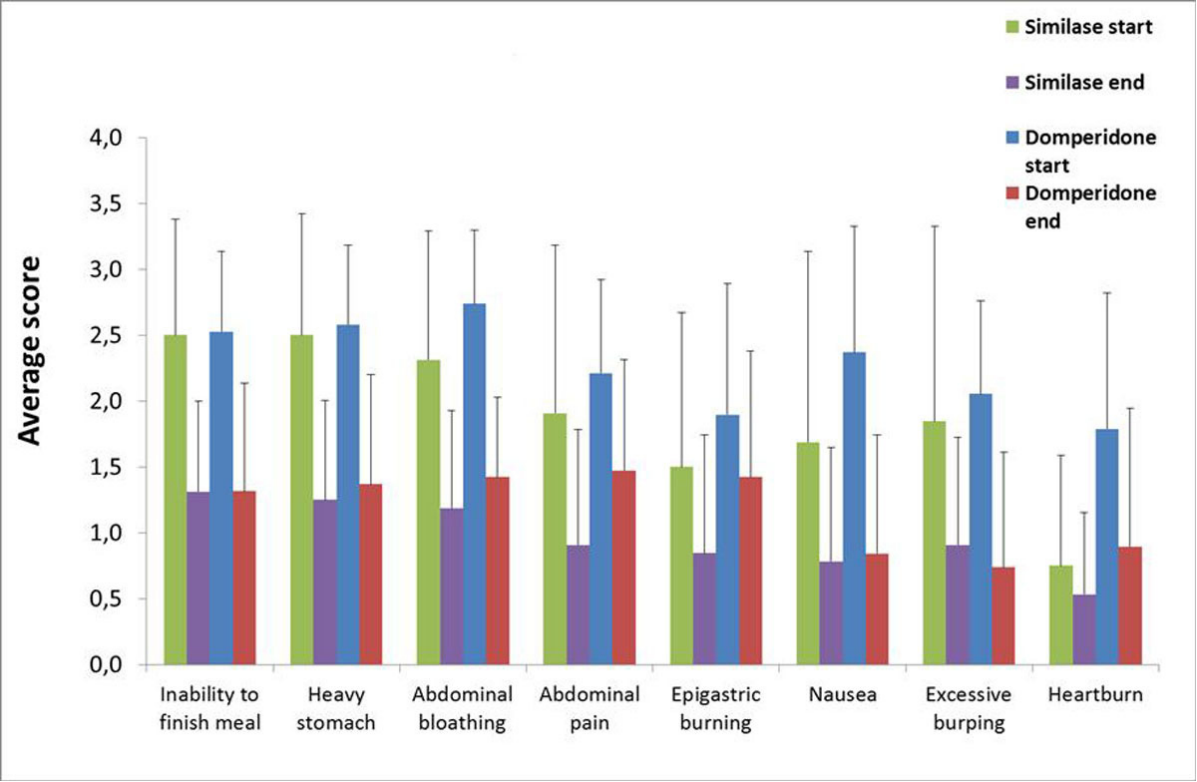
Evolution common GI-complaints.

An overview of the results is presented in Figure 1. Regarding the different gastrointestinal complaints, significant improvements of all symptoms were seen following treatment with domperidone (p<0.05) or Similase (p<0.05) as evidenced by decreased scores. After five days of treatment, Similase was significantly better in reducing abdominal pain compared to domperidone (p=0.021). For the other gastrointestinal complaints, no significant differences were seen between domperidone and Similase.

## Conclusion

This study showed that a digestive enzyme complex may offer a valuable alternative to gastroprokinetics to relieve various common gastrointestinal complaints.

